# The cross-national epidemiology of social anxiety disorder: Data from the World Mental Health Survey Initiative

**DOI:** 10.1186/s12916-017-0889-2

**Published:** 2017-07-31

**Authors:** Dan J. Stein, Carmen C. W. Lim, Annelieke M. Roest, Peter de Jonge, Sergio Aguilar-Gaxiola, Ali Al-Hamzawi, Jordi Alonso, Corina Benjet, Evelyn J. Bromet, Ronny Bruffaerts, Giovanni de Girolamo, Silvia Florescu, Oye Gureje, Josep Maria Haro, Meredith G. Harris, Yanling He, Hristo Hinkov, Itsuko Horiguchi, Chiyi Hu, Aimee Karam, Elie G. Karam, Sing Lee, Jean-Pierre Lepine, Fernando Navarro-Mateu, Beth-Ellen Pennell, Marina Piazza, Jose Posada-Villa, Margreet ten Have, Yolanda Torres, Maria Carmen Viana, Bogdan Wojtyniak, Miguel Xavier, Ronald C. Kessler, Kate M. Scott, WHO World Mental Health Survey Collaborators

**Affiliations:** 1 Department of Psychiatry and Mental Health, University of Cape Town, Cape Town, Republic of South Africa; 2 Department of Psychological Medicine, University of Otago, Dunedin, Otago New Zealand; 3 Queensland Brain Institute, University of Queensland, St Lucia, Queensland Australia; 4 Queensland Centre for Mental Health Research, The Park Centre for Mental Health, Wacol, Queensland Australia; 5 Interdisciplinary Center Psychopathology and Emotion Regulation (ICPE), University Medical Center Groningen, Groningen, Netherlands; 6 Department of Developmental Psychology, University of Groningen, Groningen, Netherlands; 7 Center for Reducing Health Disparities, UC Davis Health System, Sacramento, California USA; 8 College of Medicine, Al-Qadisiya University, Diwaniya governorate, Iraq; 9 Health Services Research Unit, IMIM-Hospital del Mar Medical Research Institute, Barcelona, Spain; 10 Pompeu Fabra University (UPF), Barcelona, Spain; 11 CIBER en Epidemiología y Salud Pública (CIBERESP), Barcelona, Spain; 12 Department of Epidemiologic and Psychosocial Research, National Institute of Psychiatry Ramón de la Fuente, Mexico City, Mexico; 13 Department of Psychiatry, Stony Brook University School of Medicine, Stony Brook, New York USA; 14 Universitair Psychiatrisch Centrum - Katholieke Universiteit Leuven (UPC-KUL), Campus Gasthuisberg, Leuven, Belgium; 15 IRCCS St John of God Clinical Research Centre//IRCCS Centro S. Giovanni di Dio Fatebenefratelli, Brescia, Italy; 16 National School of Public Health, Management and Professional Development, Bucharest, Romania; 17 Department of Psychiatry, University College Hospital, Ibadan, Nigeria; 18 Parc Sanitari Sant Joan de Déu, CIBERSAM, Universitat de Barcelona, Barcelona, Spain; 19 School of Public Health, The University of Queensland, Herston, Queensland, Australia; 20 Shanghai Mental Health Center, Shanghai, China; 21 National Center for Public Health and Analyses, Sofia, Bulgaria; 22 Center for Public Relations Strategy, Nagasaki University (Tokyo Office), Tokyo, Japan; 23 Shenzhen Institute of Mental Health & Shenzhen Kangning Hospital, Shenzhen, China; 24 Institute for Development, Research, Advocacy & Applied Care (IDRAAC), Beirut, Lebanon; 25 Department of Psychiatry and Clinical Psychology, Faculty of Medicine, Balamand University, Beirut, Lebanon; 26 Department of Psychiatry and Clinical Psychology, St George Hospital University Medical Center, Beirut, Lebanon; 27 Department of Psychiatry, Chinese University of Hong Kong, Tai Po, Hong Kong; 28 Hôpital Lariboisière Fernand Widal, Assistance Publique Hôpitaux de Paris INSERM UMR-S 1144, University Paris Diderot and Paris Descartes, Paris, France; 29 UDIF-SM, Subdirección General de Planificación, Innovación y Cronicidad, Servicio Murciano de Salud. IMIB-Arrixaca. CIBERESP-Murcia, Murcia, Spain; 30 Survey Research Center, Institute for Social Research, University of Michigan, Ann Arbor, Michigan USA; 31 Universidad Cayetano Heredia, Lima, Peru; 32 National Institute of Health, Lima, Peru; 33 Colegio Mayor de Cundinamarca University, Bogota, Colombia; 34 Trimbos-Instituut, Netherlands Institute of Mental Health and Addiction, Utrecht, Netherlands; 35 Netherlands Institute of Mental Health and Addiction, Utrecht, Netherlands; 36 Center for Excellence on Research in Mental Health, CES University, Medellin, Colombia; 37 Department of Social Medicine, Federal University of Espírito Santo, Vitoria, Brazil; 38 Centre of Monitoring and Analyses of Population Health, National Institute of Public Health-National Institute of Hygiene, Warsaw, Poland; 39 Chronic Diseases Research Center (CEDOC) and Department of Mental Health, Faculdade de Ciências Médicas, Universidade Nova de Lisboa, Campo dos Mártires da Pátria, 130, 1169-056 Lisbon, Portugal; 40 Department of Health Care Policy, Harvard Medical School, Boston, Massachusetts USA

**Keywords:** Social anxiety disorder, Social phobia, Cross-national epidemiology, World Mental Health Survey Initiative

## Abstract

**Background:**

There is evidence that social anxiety disorder (SAD) is a prevalent and disabling disorder. However, most of the available data on the epidemiology of this condition originate from high income countries in the West. The World Mental Health (WMH) Survey Initiative provides an opportunity to investigate the prevalence, course, impairment, socio-demographic correlates, comorbidity, and treatment of this condition across a range of high, middle, and low income countries in different geographic regions of the world, and to address the question of whether differences in SAD merely reflect differences in threshold for diagnosis.

**Methods:**

Data from 28 community surveys in the WMH Survey Initiative, with 142,405 respondents, were analyzed. We assessed the 30-day, 12-month, and lifetime prevalence of SAD, age of onset, and severity of role impairment associated with SAD, across countries. In addition, we investigated socio-demographic correlates of SAD, comorbidity of SAD with other mental disorders, and treatment of SAD in the combined sample. Cross-tabulations were used to calculate prevalence, impairment, comorbidity, and treatment. Survival analysis was used to estimate age of onset, and logistic regression and survival analyses were used to examine socio-demographic correlates.

**Results:**

SAD 30-day, 12-month, and lifetime prevalence estimates are 1.3, 2.4, and 4.0% across all countries. SAD prevalence rates are lowest in low/lower-middle income countries and in the African and Eastern Mediterranean regions, and highest in high income countries and in the Americas and the Western Pacific regions. Age of onset is early across the globe, and persistence is highest in upper-middle income countries, Africa, and the Eastern Mediterranean. There are some differences in domains of severe role impairment by country income level and geographic region, but there are no significant differences across different income level and geographic region in the proportion of respondents with any severe role impairment. Also, across countries SAD is associated with specific socio-demographic features (younger age, female gender, unmarried status, lower education, and lower income) and with similar patterns of comorbidity. Treatment rates for those with any impairment are lowest in low/lower-middle income countries and highest in high income countries.

**Conclusions:**

While differences in SAD prevalence across countries are apparent, we found a number of consistent patterns across the globe, including early age of onset, persistence, impairment in multiple domains, as well as characteristic socio-demographic correlates and associated psychiatric comorbidities. In addition, while there are some differences in the patterns of impairment associated with SAD across the globe, key similarities suggest that the threshold for diagnosis is similar regardless of country income levels or geographic location. Taken together, these cross-national data emphasize the international clinical and public health significance of SAD.

## Background

There is evidence from both community and clinical studies that social anxiety disorder (SAD), previously termed social phobia, is a prevalent and disabling disorder. In the National Comorbidity Survey (NCS) and National Comorbidity Survey Replication (NCS-R), SAD was one of the most common of all mental disorders (with lifetime prevalence estimates of 16% and 12.1% respectively) [1, 2]. In each of these surveys, SAD age of onset was early, comorbidity with other mental disorders was high, and subsequent impairment was notable [3, 4]. Research in clinical settings has also indicated that SAD is a prevalent and disabling condition in this context [5, 6]. Such data have been key in suggesting the clinical and public health relevance of SAD.

Nevertheless, most of the available data on the epidemiology of SAD originate from high income countries in the West. European epidemiological data have largely been consistent with US data, emphasizing the high prevalence, comorbidity, and morbidity of SAD [7]. A study using the Diagnostic Interview Schedule in four countries (USA, Canada, Korea, and Puerto Rico) found some consistent patterns, including higher rates in females and considerable comorbidity [8]. Still, many questions about the cross-national epidemiology of SAD remain unanswered. It has been suggested, for example, that anxiety disorders such as SAD are a peculiarly Western construct (in the East, for example, there may be more concern with offending others than with embarrassing oneself) [9]; from this perspective it might be hypothesized that SAD is less prevalent elsewhere, or that thresholds for SAD diagnosis differ across the globe.

Few data have systematically addressed the 30-day prevalence of SAD (which is important in establishing the prevalence at a particular point in time), whether age of onset and persistence vary across a range of different countries, whether impairment associated with SAD differs from place to place, and whether SAD treatment differs across the globe. Data on socio-demographic correlates of SAD and on comorbidity with other mental disorders have again mainly been reported in high income Western contexts. The WHO World Mental Health (WMH) Survey Initiative provides an important opportunity to investigate the epidemiology of SAD across a range of countries. In the current study, we assessed 30-day, 12-month, and lifetime SAD prevalence; age of onset; persistence; severity of role impairment associated with SAD; and treatment of SAD, across countries. In addition we investigated socio-demographic correlates of SAD, and comorbidity of SAD with other mental disorders, in the combined sample.

## Methods

### Samples

Interviews were administered in 13 regions classified by the World Bank [10] as high income (Australia, Belgium, France, Germany, Italy, Japan, New Zealand, Northern Ireland, Poland, Portugal, Spain, The Netherlands, USA), seven as upper-middle income (Brazil, Bulgaria, Colombia-Medellin, Lebanon, Mexico, Romania, South Africa), and six as low/lower-middle income (Colombia, Iraq, Nigeria, Peru, People’s Republic of China [PRC], Ukraine). Classified by region, surveys are from Africa (Nigeria, South Africa), the Americas (Brazil, Colombia, Mexico, Peru, USA), Eastern Europe (Bulgaria, Poland, Romania, Ukraine), Western Europe (Belgium, France, Germany, Italy, Northern Ireland, Portugal, Spain, The Netherlands), Western Pacific (Australia, Japan, New Zealand, PRC), and Eastern Mediterranean (Iraq, Lebanon).

All but ten surveys were based on area probability household samples representative of the entire nation (see Table 1 for survey details). The exceptions were surveys of all urbanized areas in three countries (Colombia, Mexico, Peru), of a specific region in two countries (Colombia-Medellin, Spain-Murcia), of specific metropolitan areas in three countries (São Paulo in Brazil; a series of cities in Japan; Beijing, Shanghai and Shen Zhen in PRC) and of selected states in one country (Nigeria). Respondents had to be at least 18 years of age in most countries (20 in Japan). Five surveys (Colombia, Colombia-Medellin, Mexico, Peru, Poland) had an upper age limit (64 or 65), and one (Australia) had an upper age limit of 85.

**Table 1:** World Mental Health sample characteristics by World Bank income categories

Country	Survey	Sample characteristics	Field dates	Age range^b^	Sample size		Response rate (%)
					Part 1	Part 2 subsample	
Low/lower-middle income countries^a^							
Colombia	NSMH	All urban areas of the country (approximately 73% of the total national population)	2003	18–65	4426	2381	87.7
Iraq	IMHS	Nationally representative	2006–2007	18+	4332	4332	95.2
Nigeria	NSMHW	21 of the 36 states in the country, representing 57% of the national population. The surveys were conducted in Yoruba, Igbo, Hausa and Efik languages	2002–2003	18+	6752	2143	79.3
Peru	EMSMP	Five urban areas of the country (approximately 38% of the total national population)	2004–2005	18–65	3930	1801	90.2
PRC Beijing/Shanghai	B-WMH S-WMH	Beijing and Shanghai metropolitan areas	2002–2003	18+	5201	1628	74.7
PRC Shen Zhen	Shenzhen	Shen Zhen metropolitan area. Included temporary residents as well as household residents	2006–2007	18+	7132	2475	80.0
Ukraine	CMDPSD	Nationally representative	2002	18+	4725	1720	78.3
Upper-middle income countries^a^							
Brazil	São Paulo Megacity	São Paulo metropolitan area	2005–2007	18+	5037	2942	81.3
Bulgaria	NSHS	Nationally representative	2003–2007	18+	5318	2233	72.0
Colombia (Medellin)^c^	MMHHS	Medellin metropolitan area	2011–2012	18–65	3261	1673	97.2
Lebanon	LEBANON	Nationally representative	2002–2003	18+	2857	1031	70.0
Mexico	M-NCS	All urban areas of the country (approximately 75% of the total national population)	2001–2002	18–65	5782	2362	76.6
Romania	RMHS	Nationally representative	2005–2006	18+	2357	2357	70.9
South Africa	SASH	Nationally representative	2003–2004	18+	4315	4315	87.1
High income countries^a^							
Australia	SMHWB	Nationally representative	2007	18–85	8463	8463	60.0
Belgium	ESEMeD	Nationally representative	2001–2002	18+	2419	1043	50.6
France	ESEMeD	Nationally representative	2001–2002	18+	2894	1436	45.9
Germany	ESEMeD	Nationally representative	2002–2003	18+	3555	1323	57.8
Italy	ESEMeD	Nationally representative	2001–2002	18+	4712	1779	71.3
Japan	WMHJ	Eleven metropolitan areas	2002–2006	20+	4129	1682	55.1
New Zealand	NZMHS	Nationally representative	2003–2004	18+	12790	7312	73.3
Northern Ireland	NISHS	Nationally representative	2004–2007	18+	4340	1986	68.4
Poland	EZOP	Nationally representative	2010–2011	18–64	10081	4000	50.4
Portugal	NMHS	Nationally representative	2008–2009	18+	3849	2060	57.3
Spain	ESEMeD	Nationally representative	2001–2002	18+	5473	2121	78.6
Spain (Murcia)	PEGASUS-Murcia	Murcia region	2010–2012	18+	2621	1459	67.4
The Netherlands	ESEMeD	Nationally representative	2002–2003	18+	2372	1094	56.4
USA	NCS-R	Nationally representative	2002–2003	18+	9282	5692	70.9
Total					142,405	74,843	
Weighted average response rate (%)							69.4

^a^The World Bank. (2008). Data and Statistics. Accessed May 12, 2009 at: http://go.worldbank.org/D7SN0B8YU0

^b^For the purposes of cross-national comparisons we limit the sample to those 18+

^c^The newer Colombian survey in Medellin classified Colombia as an upper-middle income country (due to a change of classification by the World Bank), although in the original survey Colombia was classified as a low/lower-middle income country

ESEMeD (The European Study Of The Epidemiology Of Mental Disorders); NHS (Israel National Health Survey); WMHJ 2002-2006 (World Mental Health Japan Survey); NZMHS (New Zealand Mental Health Survey); NCS-R (The USA National Comorbidity Survey Replication); NSMH (The Colombian National Study of Mental Health); WMHI (World Mental Health India); LEBANON (Lebanese Evaluation of the Burden of Ailments and Needs of the Nation); M-NCS (The Mexico National Comorbidity Survey); SASH (South Africa Stress and Health Study); CMDPSD (Comorbid Mental Disorders during Periods of Social Disruption)

Interviews were conducted face to face in respondent homes after obtaining informed consent. Human Subjects Committees monitored the surveys and approved recruitment and consent procedures in each country. Other than in Australia, Iraq, Romania, and South Africa, where all respondents were administered the full interview, internal subsampling was used to reduce respondent burden by dividing the interview into two parts. Part 1 assessed core disorders, including SAD, and was administered to all respondents. Part 2 included additional disorders and correlates and was administered to all Part 1 respondents who met criteria for any lifetime Part 1 disorder plus a probability subsample of other respondents. Part 1 data were weighted to adjust for differential probabilities of selection and to match population distributions on census socio-demographic and geographic distributions. Part 2 data were additionally weighted for the under-sampling of Part 1 respondents without core disorders. Response rates range from a low of 45.9% (France) to 97.2% (Colombia-Medellin) (69.4% weighted average) (Table 1). Technical details about WMH sample design are presented elsewhere [11].

### Measures

The WMH interviews assess prevalence and a wide range of predictors and consequences of numerous anxiety, mood, impulse control, and substance use disorders [12]. The full text of the interview schedule is available at www.hcp.med.harvard.edu/wmh. The WMH interview schedule was developed in English and translated into other languages using a standardized WHO translation, back-translation, and harmonization protocol described elsewhere [13]. Consistent interviewer training and quality control monitoring procedures were used in all surveys to facilitate cross-national comparison [14]. The following sections emphasize the measures considered in the current report.

#### Mental disorders

SAD and other Diagnostic and Statistical Manual of Mental Disorders (DSM)-IV anxiety (i.e., panic disorder with or without agoraphobia, agoraphobia without panic disorder, generalized anxiety disorder, specific phobia, post traumatic stress disorder, and separation anxiety disorder), mood (i.e., major depressive episode, bipolar disorder), impulse control (i.e., intermittent explosive disorder, bulimia nervosa, binge eating disorder, oppositional defiant disorder, conduct disorder, attention deficit disorder), and substance use disorders (i.e., alcohol abuse and drug abuse with or without dependence) were assessed using Version 3.0 of the WHO Composite International Diagnostic Interview (CIDI 3.0) [15], a fully structured lay-administered interview. Respondents were administered the full SAD section if they endorsed a diagnostic stem question for one or more performance or interactional fears described as excessive and causing substantial distress or avoidance. The SAD section screened for lifetime experiences of shyness, fear, and discomfort associated with each of 14 social situations. Respondents endorsing one or more such questions were asked about all DSM-IV criteria. Age of onset (AOO) of each disorder was assessed using special probing techniques shown experimentally to improve recall accuracy [16]. CIDI diagnoses were compared to blinded clinical diagnoses using the Structured Clinical Interview for DSM-IV (SCID) [17] in probability subsamples of WMH respondents from France, Italy, Spain, and the USA. As detailed elsewhere, good CIDI-SCID diagnostic concordance was found for SAD — area under the curve (AUC) = 0.67 — and most other DSM-IV/CIDI disorders [18].

#### Impairment

The Sheehan Disability Scale (SDS) [19] was used to assess recent impairment in role functioning in each of four domains (home, work, relationship, and social) in respondents with a 12-month SAD diagnosis. The response scale is from 0 to 10, with severe impairment in a specific role domain defined as a score ≥7. In addition, respondents were asked how many days in the past year they were unable to work or carry out their normal activities due to their disorder (days out of role).

#### Treatment

The 12-month treatment was assessed by asking respondents if they had seen any of a list of professionals for problems with emotions, nerves, mental health, or alcohol or drug use, including both inpatient and outpatient care. Sectors included were as follows: specialty mental health (e.g., psychiatrist and non-psychiatrist mental health specialist), general medical (e.g., general practitioner), human services sector (e.g., religious advisor), and complementary and alternative medicine (e.g., herbalist or homeopath).

#### Demographic factors

We examined age (18–29, 30–44, 45–59, 60+), time since onset, gender, employment status (student, homemaker, retired, other, employed), marital status (never married, divorced/separated/widowed, currently married), education level (no education, some primary, finished primary, some secondary, finished secondary, some college, finished college), and household income (low, low average, high average, and high, which were based on country-specific quartiles of gross household earnings in the past 12 months) [20].

### Statistical analysis

Cross-tabulations were used to calculate prevalence, impairment, comorbidity, and treatment. Significance was calculated using Wald and McNemar’s chi-square tests. Survival analysis was used to estimate AOO and projected lifetime risk, as the young age of many respondents biases the AOO distribution downwards. The actuarial method implemented in SAS 9.4 (PROC LIFETEST) was used to generate the AOO curves. Logistic regression and survival analyses were used to examine socio-demographic correlates. Because the data were weighted and clustered, the Taylor series linearization method [21] implemented in the SUDAAN software package 11.0 [22] was used to estimate design-based standard errors. Statistical significance was consistently evaluated using two-sided tests, with *P* < 0.05 considered significant.

## Results

### Prevalence

On average, the estimated lifetime, 12-month, and 30-day prevalence is highest in high income countries (5.5%, 3.1%, 1.7%), intermediate in upper-middle income countries (2.9%, 2.1%, 1.3%), and lowest in low/lower-middle income countries (1.6%, 1.0%, 0.5%) (Table 2). Prevalence rates are highest in the Americas and the Western Pacific region, and lowest in Africa and the Eastern Mediterranean. Across all countries, SAD is a prevalent disorder (4.0%, 2.4%, 1.3%). Comparison of lifetime, 12-month, and 30-day prevalence across all countries, across different income groups, and across different regional groups all reached significance (*P* < 0.001) (Table 2).

**Table 2:** Prevalence of DSM-IV social anxiety disorder (SAD) in the World Mental Health surveys

Country	Lifetime prevalence		12-month prevalence		30-day prevalence		12-month prevalence of SAD among lifetime cases		30-day prevalence of SAD among 12-month cases		Sample size used
	%	SE	%	SE	%	SE	%	SE	%	SE	
Low/lower-middle income countries	1.6	0.1	1.0	0.1	0.5	0.0	62.6	2.5	52.0	3.4	36,498
Colombia	5.0	0.5	2.9	0.3	1.6	0.3	58.0	4.6	54.9	6.1	4426
Iraq	0.8	0.2	0.7	0.2	0.5	0.2	86.0	7.5	72.0	6.9	4332
Nigeria	0.2	0.1	0.2	0.1	0.1	0.1	96.3	3.9	83.3	11.7	6752
Peru	2.6	0.3	1.4	0.1	0.5	0.1	54.2	3.2	35.5	6.8	3930
PRC China	0.5	0.1	0.4	0.1	0.2	0.1	66.6	11.9	52.8	13.7	5201
PRC Shen Zhen	0.9	0.2	0.7	0.1	0.2	0.1	76.5	6.0	29.3	9.9	7132
Ukraine	2.6	0.2	1.5	0.2	1.0	0.2	59.9	4.9	62.3	7.8	4725
Upper-middle income countries	2.9	0.1	2.1	0.1	1.3	0.1	72.4	2.1	61.4	2.6	28,927
Brazil	5.6	0.4	3.9	0.3	2.7	0.3	70.8	4.5	67.5	5.2	5037
Bulgaria	0.8	0.2	0.6	0.2	0.4	0.1	74.7	7.0	58.9	9.4	5318
Colombia (Medellin)	4.6	0.5	3.8	0.5	2.2	0.4	82.7	3.8	58.3	6.5	3261
Lebanon	1.9	0.4	1.3	0.3	0.8	0.2	67.0	7.0	61.3	9.4	2857
Mexico	2.9	0.2	2.0	0.2	1.1	0.2	69.4	4.0	53.4	4.9	5782
Romania	1.3	0.3	1.0	0.2	0.6	0.2	74.7	8.3	60.1	12.2	2357
South Africa	2.8	0.4	1.9	0.3	1.2	0.2	68.7	5.8	64.4	5.6	4315
High income countries	5.5	0.1	3.1	0.1	1.7	0.1	57.3	1.0	53.1	1.2	76,980
Australia	8.5	0.4	4.2	0.3	1.9	0.2	49.8	2.9	44.7	3.3	8463
Belgium	2.0	0.4	1.2	0.2	0.7	0.2	59.8	7.2	58.4	13.5	2419
France	4.3	0.5	2.6	0.4	1.8	0.3	59.3	5.2	71.8	6.7	2894
Germany	2.5	0.3	1.5	0.2	1.0	0.2	60.4	6.0	63.7	7.7	3555
Italy	1.9	0.2	1.1	0.2	0.6	0.1	60.0	5.4	52.8	8.6	4712
Japan	1.4	0.2	0.7	0.2	0.5	0.1	51.9	8.1	68.4	9.3	4129
New Zealand	9.5	0.3	5.3	0.3	2.8	0.2	56.0	1.8	52.5	2.5	12,790
Northern Ireland	6.0	0.4	4.0	0.3	2.5	0.3	65.8	2.9	63.4	4.6	4340
Poland	1.4	0.1	0.9	0.1	0.5	0.1	63.4	3.8	55.1	4.5	10,081
Portugal	4.7	0.5	3.1	0.4	1.7	0.2	67.1	3.9	54.2	4.8	3849
Spain	1.2	0.2	0.7	0.1	0.4	0.1	56.3	6.9	58.6	12.4	5473
Spain (Murcia)	1.7	0.2	1.2	0.2	0.9	0.2	67.7	11.0	74.4	10.3	2621
The Netherlands	2.6	0.4	1.3	0.3	1.0	0.3	50.8	9.3	73.9	8.1	2372
USA	12.1	0.4	7.1	0.3	3.5	0.2	58.8	1.7	48.9	1.9	9282
All countries combined	4.0	0.1	2.4	0.1	1.3	0.0	60.2	0.8	54.5	1.0	142,405
WHO regions^a^											
Region of the Americas	6.4	0.2	4.0	0.1	2.1	0.1	62.8	1.3	53.1	1.6	31,718
African region	1.2	0.2	0.9	0.1	0.6	0.1	71.1	5.5	66.7	5.3	11,067
Western Pacific region	5.5	0.2	3.0	0.1	1.5	0.1	54.5	1.5	49.4	1.9	37,715
Eastern Mediterranean region	1.2	0.2	0.9	0.2	0.6	0.1	74.2	5.7	66.0	6.1	7189
Western European region	3.0	0.1	1.9	0.1	1.2	0.1	62.4	1.8	62.3	2.5	32,235
Eastern European region	1.5	0.1	1.0	0.1	0.6	0.1	64.7	2.7	58.6	3.7	22,481
Comparison between countries^b^	χ^2^_27_ = 78.6*, *P* < 0.001		χ^2^_27_ = 46.3*, *P* < 0.001		χ^2^_27_ = 25.7*, *P* < 0.001		χ^2^_27_ = 3.3*, *P* < 0.001		χ^2^_27_ = 2.4*, *P* < 0.001		
Comparison between low, middle, and high income country groups^b^	χ^2^_2_ = 387.5*, *P* < 0.001		χ^2^_2_ = 224.2*, *P* < 0.001		χ^2^_2_ = 121.7*, *P* < 0.001		χ^2^_2_ = 21.3*, *P* < 0.001		χ^2^_2_ = 4.5*, *P* = 0.01		
Comparison between WHO regions^b^	χ^2^_5_ = 207.5*, *P* < 0.001		χ^2^_5_ = 118.4*, *P* < 0.001		χ^2^_5_ = 53.2*, *P* < 0.001		χ^2^_5_ = 6.1*, *P* < 0.001		χ^2^_5_ = 5.2*, *P* < 0.001		

*Significant at the 0.05 level

^a^ *Region of the Americas* (Colombia, Mexico, Brazil, Peru, USA, Medellin); *African region* (Nigeria, South Africa); *Western Pacific region* (PRC Shen Zhen, PRC Beijing and Shanghai, Japan, Australia, New Zealand); *Eastern Mediterranean region* (Iraq, Lebanon); *Western European region* (Belgium, France, Germany, Italy, The Netherlands, Spain, Northern Ireland, Portugal, Murcia); *Eastern European region* (Romania, Bulgaria, Poland, Ukraine)

^b^Chi-square test of homogeneity to determine if there is variation in prevalence estimates across countries

*SE* standard error

The ratio of the 12-month prevalence to lifetime prevalence is an indirect indicator of disorder persistence. This ratio is lowest in high income countries (57.3%) and the Western Pacific (54.5%), and highest in upper-middle income countries (72.4%), Africa (71.1%), and the Eastern Mediterranean (74.2%). Across all countries, SAD is a persistent disorder (60.2%). The ratio of the 30-day prevalence to 12-month prevalence is an indirect indicator of episode persistence among those with recent disorder. This ratio is again lowest in the Western Pacific (49.4%), and highest in upper-middle income countries (61.4%), Africa (66.7%), and the Eastern Mediterranean (66.0%). Comparison of disorder and episode persistence across all countries, across different income groups, and across different regional groups all reached significance (*P* < 0.001) (Table 2).

### Age of onset

**Fig. 1:**
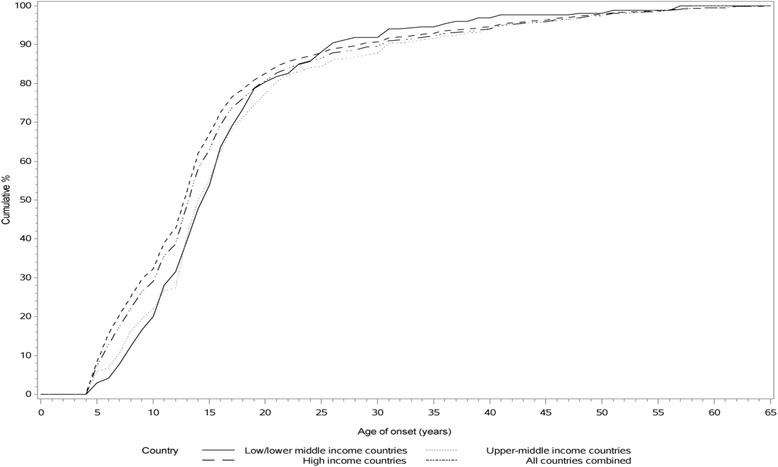
Age of onset of SAD by country income level

Table 3 and Fig. 1 indicate that the median estimated AOO is similar for high income, upper-middle income, and low/lower-middle income countries. Across all countries, the risk period for onset of SAD ranges from the mid-late adolescence to the early 40s. In high income countries, the earliest median AOO estimates occurr in Poland (50% by age 11), whereas the latest are in The Netherlands (50% by age 17). In upper-middle countries, the earliest median AOO estimates are in Colombia (50% by age 13), and the latest in South Africa (50% by age 26). In low/lower-middle income countries, the earliest median AOO estimates are in Nigeria (50% by age 11), and the latest in Peru (50% by age 16). Projected lifetime risk for SAD across the globe is 4.4%.

**Table 3:** Age at selected percentiles on the standardized age of onset distributions of DSM-IV SAD with projected lifetime risk at age 75

Country	Ages at selected percentiles								Lifetime prevalence of SAD		Projected risk at age 75	
	5	10	25	50	75	90	95	99	%	SE	%	SE
Low/lower-middle income countries	7	8	11	15	19	26	36	57	1.6	0.1	1.7	0.1
Colombia^a^	6	8	11	15	19	26	31	39	5.0	0.5	5.3	0.5
Iraq	7	9	13	14	18	23	36	36	0.8	0.2	0.8	0.2
Nigeria	7	7	7	11	19	23	24	24	0.2	0.1	0.2	0.1
Peru^a^	9	10	13	16	19	27	34	41	2.6	0.3	2.7	0.3
PRC China	8	12	14	14	17	19	37	37	0.5	0.1	0.5	0.1
PRC Shen Zhen	5	7	11	14	18	26	31	41	0.9	0.2	1.0	0.2
Ukraine	7	8	11	14	16	25	37	57	2.6	0.2	2.9	0.3
Upper-middle income countries	5	7	11	15	20	36	49	67	2.9	0.1	3.4	0.2
Brazil	5	7	11	14	17	29	41	54	5.6	0.4	6.1	0.4
Bulgaria	8	8	11	14	18	24	31	38	0.8	0.2	0.9	0.2
Colombia (Medellin)^a^	5	5	8	13	16	21	31	41	4.6	0.5	4.7	0.5
Lebanon	6	7	11	14	18	20	26	30	1.9	0.4	2.0	0.4
Mexico^a^	6	7	11	15	19	26	40	54	2.9	0.2	3.2	0.3
Romania	10	13	14	21	36	58	58	58	1.3	0.3	1.8	0.4
South Africa	11	13	16	26	49	67	67	67	2.8	0.4	4.7	1.2
High income countries	5	6	9	13	17	29	42	59	4.0	0.1	6.0	0.1
Australia	5	6	9	14	20	37	46	68	8.5	0.4	9.6	0.5
Belgium	5	5	7	13	17	25	36	36	2.0	0.4	2.2	0.4
France	7	8	11	14	20	31	45	57	4.3	0.5	4.9	0.5
Germany	7	9	11	14	35	50	62	62	2.5	0.3	3.0	0.5
Italy	5	7	13	15	20	28	36	56	1.9	0.2	2.0	0.3
Japan	5	5	9	13	16	29	43	48	1.4	0.2	1.6	0.2
New Zealand	5	6	8	13	17	27	38	57	9.5	0.3	10.4	0.4
Northern Ireland	5	6	10	14	20	40	49	54	6.0	0.4	7.1	0.5
Poland^b^	5	5	8	11	14	17	19	21	1.4	0.1	1.4	0.1
Portugal	5	5	9	14	18	29	43	61	4.7	0.5	5.2	0.5
Spain	5	5	9	13	19	22	48	48	1.2	0.2	1.3	0.2
Spain (Murcia)	5	5	5	13	18	33	37	40	1.7	0.2	1.9	0.3
The Netherlands	5	7	11	17	29	41	49	52	2.6	0.4	3.1	0.5
USA	5	6	8	13	15	23	32	51	12.1	0.4	13.0	0.5
All countries combined	5	6	9	14	18	31	44	62	4.0	0.1	4.4	0.1
WHO regions												
Region of the Americas	5	6	9	13	17	26	36	52	6.4	0.2	6.9	0.2
African region	7	13	15	23	47	67	67	67	1.2	0.2	2.0	0.5
Western Pacific region	5	6	9	14	18	33	46	66	5.5	0.2	6.1	0.2
Eastern Mediterranean region	6	8	11	14	18	23	26	36	1.2	0.2	1.3	0.2
Western European region	5	6	10	14	20	36	45	61	3.0	0.1	3.4	0.1
Eastern European region	5	7	9	13	17	24	38	58	1.5	0.1	1.7	0.1

^a^The projected risk for these countries is at age 65 because the age range of these surveys is between 18–65

^b^The projected risk for this country is at age 64 because the age range of this survey is between 18–64

*SE* standard error

### Impairment

SAD is associated with substantial impairment in multiple domains of role functioning in the WMH data (Table 4) and with a mean number of days out of work of 24.7 (1.8) in the past year (Appendix 1: Table 8). However, in most countries, the proportion of respondents with 12-month SAD and severe role impairment (SDS score of 7–10) is higher in the domains of relationships and social situations than in the domains of home and work. Furthermore, in most countries, between one-third and one-half of respondents with 12-month SAD have severe role impairment in at least one domain. Notably, there are no significant differences between low, middle, and high income groups, or between different WHO regions, in the proportion of respondents with severe role impairment in at least one domain.

**Table 4:** Severity of role impairment (Sheehan Disability Scale: SDS) associated with 12-month SAD, by country

Country	Proportion with severe role impairment (SDS score: 7–10)										Number of 12-month cases
	Home		Work		Relationship		Social		Any^a^		
	%	SE	%	SE	%	SE	%	SE	%	SE	
Low/lower-middle income countries^c,d,e,g^	9.3	1.6	14.1	2.4	18.0	2.6	21.2	2.8	34.3	3.2	349
Colombia^c,d,e,g,h^	8.1	2.3	18.1	5.2	22.5	4.9	32.3	5.0	43.2	5.3	133
Iraq^f^	18.0	9.2	9.0	5.4	31.6	12.7	22.7	8.3	48.0	12.6	28
Nigeria	7.8	7.8	28.2	15.7	24.1	13.9	24.1	13.9	36.3	17.4	9
Peru	13.7	4.7	13.4	5.2	11.7	4.0	20.6	7.7	33.0	7.9	51
PRC China	4.9	4.8	4.6	4.6	4.6	4.6	17.4	12.1	26.9	13.0	16
PRC Shen Zhen	2.1	1.9	1.4	1.2	1.2	1.2	6.1	3.5	9.4	4.2	45
Ukraine^d,h^	11.5	4.1	18.4	5.6	23.1	5.8	12.2	4.7	33.0	6.4	67
Upper-middle income countries^c,d,e,f,g^	12.7	1.8	17.0	2.5	28.5	2.2	28.5	2.2	39.3	2.6	601
Brazil^c,d,e,g^	13.9	4.0	20.5	6.1	25.8	3.5	27.7	3.9	36.7	4.7	186
Bulgaria^d,f^	5.3	2.9	2.5	1.0	23.2	11.0	10.0	4.7	25.8	10.8	27
Colombia (Medellin)^c,d,e,f,g^	12.5	4.0	19.8	4.6	33.5	6.1	33.6	6.0	43.2	6.1	110
Lebanon^d,e,f,g^	14.1	6.4	7.9	5.5	43.7	10.0	33.8	9.8	45.8	9.6	35
Mexico^d,e,f,g^	7.3	2.0	11.9	3.2	23.4	3.7	28.1	4.2	35.2	4.4	134
Romania	26.0	10.9	31.5	11.9	40.4	12.9	32.0	11.0	56.2	9.8	22
South Africa^d^	16.6	5.2	17.9	6.0	27.4	6.1	28.1	5.9	43.6	8.1	87
High income countries^c,d,e,f,g,h^	11.0	0.7	16.8	0.8	23.6	1.0	29.8	1.1	37.7	1.1	2510
Australia^c,d,e,f,g,h^	17.2	2.7	24.3	2.8	37.2	3.9	43.1	4.2	50.1	4.0	381
Belgium^c,d,e^	9.6	6.9	28.1	10.7	37.0	13.1	38.4	8.8	54.9	8.3	28
France^e,g^	9.9	5.1	11.0	4.3	17.5	4.0	24.0	5.3	32.9	5.9	72
Germany^c,d,e,g^	4.0	3.0	14.1	4.9	20.0	5.9	28.0	7.9	42.2	7.9	58
Italy^f^	15.9	6.1	7.9	3.9	23.3	6.0	17.1	6.3	33.1	6.9	53
Japan^c,d^	6.5	5.9	26.2	8.3	20.4	8.0	25.7	9.4	37.8	8.8	25
New Zealand^c,d,e,f,g,h^	6.1	1.1	12.3	1.3	18.8	1.9	26.7	2.1	32.5	2.1	720
Northern Ireland^d,e,g,h^	19.6	2.7	24.7	3.0	31.4	3.4	41.4	4.3	52.3	4.1	183
Poland	14.2	4.6	21.3	4.8	18.6	4.5	21.4	5.2	32.4	5.6	91
Portugal^c,d,e,g^	7.2	2.1	13.4	2.5	15.8	2.7	19.4	3.2	25.1	3.9	124
Spain	8.2	5.4	15.6	7.3	21.2	9.8	17.0	8.2	26.3	10.5	33
Spain (Murcia)^c,d,e,f,g^	25.8	8.9	41.7	12.7	67.2	11.0	62.4	6.8	71.6	9.4	33
The Netherlands^c^	41.9	11.2	56.8	12.7	46.8	13.9	56.1	11.1	63.6	12.0	30
USA^c,d,e,f,g,h^	10.9	1.3	15.4	1.4	22.6	1.6	28.8	1.4	36.5	1.7	679
All countries combined^c,d,e,f,g,h^	11.1	0.7	16.5	0.8	23.9	0.9	28.6	0.9	37.6	1.0	3460
WHO regions											
Region of the Americas^c,d,e,f,g,h^	11.0	1.0	16.5	1.4	23.7	1.3	29.0	1.3	37.5	1.5	1293
African Region^d,e^	15.6	4.7	19.1	5.6	27.0	5.6	27.6	5.4	42.7	7.4	96
Western Pacific region^c,d,e,f,g,h^	8.1	1.0	14.2	1.1	21.4	1.6	28.7	1.8	34.9	1.8	1187
Eastern Mediterranean Region^d,e,f,g^	15.8	5.4	8.4	3.9	38.4	7.9	28.9	7.0	46.8	7.7	63
Western European Region^c,d,e,f,g,h^	14.4	1.7	20.2	2.0	26.7	2.0	31.4	2.2	41.7	2.4	614
Eastern European Region^c,d,h^	13.2	2.6	18.3	3.1	23.4	3.6	17.6	3.1	34.2	3.7	207
Comparison between countries^b^	χ^2^_27_ = 2.3*, *P* < 0.001		χ^2^_27_ = 2.9*, *P* < 0.001		χ^2^_27_ = 3.0*, *P* < 0.001		χ^2^_27_ = 2.8*, *P* < 0.001		χ^2^_27_ = 2.6*, *P* < 0.001		
Comparison between low, middle, and high income country groups^b^	χ^2^_2_ = 1.0, *P* = 0.371		χ^2^_2_ = 0.6, *P* = 0.561		χ^2^_2_ = 4.7*, *P* = 0.008		χ^2^_2_ = 4.1*, *P* = 0.016		χ^2^_2_ = 0.8, *P* = 0.463		
Comparison between WHO regions^b^	χ^2^_5_ = 2.9*, *P* = 0.013		χ^2^_5_ = 2.3*, *P* = 0.042		χ^2^_5_ = 1.5, *P* = 0.180		χ^2^_5_ = 2.7*, *P* = 0.020		χ^2^_5_ = 1.5, *P* = 0.175		

*Significant at the 0.05 level

^a^Highest severity category across four SDS role domains

^b^Chi-square test of homogeneity to determine if there is variation in impairment severity across countries. McNemar’s chi-square test to determine if there is a significant difference for ^c^home vs work impairment, ^d^home vs relationship impairment, ^e^home vs social impairment, ^f^work vs relationship impairment, ^g^work vs social impairment, ^h^relationship vs social impairment for each row entry. For example, ^c^for Colombia indicates that the proportion with severe impairment associated with social anxiety disorder is significantly higher for work than home

However, there are significant differences across countries in proportion of 12-month SAD respondents with severe role impairment in any of the domains (ranging from 9.4% in PRC Shen Zhen to 71.6% in Spain-Murcia) (Table 4), and there are also some differences in specific domains across country, income region, and WHO region. The proportion of respondents with severe home impairment varies significantly by country and by WHO region; it is lowest in PRC Shen Zhen (2.1%) and the Western Pacific (8.1%), and highest in The Netherlands (41.9%) and the Eastern Mediterranean (15.8%). The proportion of respondents with severe work impairment varies significantly by country and by WHO region; it is lowest in the PRC Shen Zhen (1.4%) and the Eastern Mediterranean (8.4%), and highest in the Netherlands (56.8%) and Western Europe (20.2%). The proportion of respondents with severe relationship impairment varies significantly by country and by income region (lowest in low/lower-middle income countries, i.e., 18%, and highest in upper-middle income countries, i.e., 28.5%). The proportion of respondents with severe social impairment varies by country, by WHO region (lowest in Eastern Europe, i.e., 17.6%, highest in Western Europe, i.e., 31.4%), and by income region (lowest in low/lower-middle income, i.e., 21.2%, highest in high income, i.e., 29.8%).

### Socio-demographic correlates

Table 5 shows the bivariate associations of the socio-demographic characteristics with SAD in the combined sample. Both 30-day and lifetime risk of SAD are associated with younger AOO, female gender, not being employed, being unmarried (never married or divorced/widowed/separated), lower educational status, and low household income. SAD recurrence (as indicated by 12-month SAD in lifetime cases) is associated with female gender, earlier AOO, and being unmarried — while persistence (as indicated by 30-day SAD in 12-month cases) is associated with female gender but not with earlier AOO or marital status. SAD recurrence is particularly highly associated with lower education (with no education having an odds ratio [OR] of 5.6, confidence interval [CI] 2.2–14.4), SAD persistence is particularly associated with being a student (OR of 2.1, CI 1.4–3.0), and both recurrence and persistence are associated with being a homemaker. Socio-demographic correlates are similar across countries for the most part, but also demonstrate some differences (Appendix 2: Table 9, Appendix 3: Table 10, and Appendix 4: Table 11).

**Table 5:** Bivariate associations between socio-demographics correlates and DSM-IV social anxiety disorder (all countries combined)

Correlates	30-day Social Anxiety Disorder^a^		Lifetime Social Anxiety Disorder^b^		12-month Social Anxiety Disorder among lifetime cases^c^		30-day Social Anxiety Disorder among 12-month cases^c^	
	OR	(95% CI)	OR	(95% CI)	OR	(95% CI)	OR	(95% CI)
Age-cohort								
18-29	3.2*	(2.6-3.9)	3.6*	(3.2-4.0)				
30-44	2.8*	(2.3-3.4)	2.9*	(2.6-3.2)				
45-59	2.5*	(2.0-3.1)	2.4*	(2.1-2.6)				
60+	1.0		1.0					
Age-cohort difference^d^	χ^2^_3_ = 145.4*, *P* < .001		χ^2^_3_ = 547.3*, *P* < .001					
Age of onset								
Early					1.5*	(1.2-1.8)	1.0	(0.7-1.2)
Early-average					1.4*	(1.1-1.7)	0.9	(0.7-1.2)
Late-average					1.1	(0.9-1.3)	0.9	(0.8-1.2)
Late					1.0		1.0	
Age of onset difference^d^					χ^2^_3_ = 15.4*, *P* = 0.002		χ^2^_3_ = 0.5, *P* = 0.926	
Time since onset (Continuous)					0.98*	(0.98-0.99)	1.01*	(1.00-1.01)
					χ^2^_1_ = 63.1*, *P* < .001		χ^2^_1_ = 5.0*, *P* = 0.025	
Gender								
Female	1.7*	(1.5-1.9)	1.3*	(1.2-1.4)	1.3*	(1.2-1.5)	1.2*	(1.0-1.4)
Male	1.0		1.0		1.0		1.0	
Gender difference^d^	χ^2^_1_ = 65.3*, *P* < .001		χ^2^_1_ = 61.5*, *P* < .001		χ^2^_1_ = 15.7*, *P* < .001		χ^2^_1_ = 5.9*, *P* = 0.015	
Employment status								
Student	1.4*	(1.1-1.9)	1.2	(1.0-1.4)	1.1	(0.8-1.6)	2.1*	(1.4-3.0)
Homemaker	1.5*	(1.3-1.7)	1.2*	(1.1-1.3)	1.4*	(1.1-1.7)	1.4*	(1.1-1.8)
Retired	0.6*	(0.5-0.8)	0.9	(0.7-1.0)	1.0	(0.7-1.3)	0.9	(0.6-1.3)
Other	1.8*	(1.5-2.1)	1.5*	(1.3-1.6)	2.0*	(1.6-2.6)	1.0	(0.8-1.3)
Employed	1.0		1.0		1.0		1.0	
Employment status difference^d^	χ^2^_4_ = 81.8*, *P* < .001		χ^2^_4_ = 63.6*, *P* < .001		χ^2^_4_ = 36.9*, *P* < .001		χ^2^_4_ = 20.4*, *P* < .001	
Marital status								
Never married	1.2*	(1.1-1.4)	1.4*	(1.3-1.5)	1.3*	(1.1-1.6)	1.0	(0.8-1.2)
Divorced/separated/widowed	1.5*	(1.3-1.7)	1.3*	(1.2-1.5)	1.4*	(1.1-1.6)	1.0	(0.8-1.3)
Currently married	1.0		1.0		1.0		1.0	
Marital status difference^d^	χ^2^_2_ = 26.6*, *P* < .001		χ^2^_2_ = 75.7*, *P* < .001		χ^2^_2_ = 18.4*, *P* < .001		χ^2^_2_ = 0.2, *P* = 0.887	
Education level								
No education	1.3	(0.8-2.2)	0.8	(0.6-1.2)	5.6*	(2.2-14.4)	1.2	(0.6-2.6)
Some primary	1.8*	(1.3-2.4)	1.1	(0.9-1.3)	3.0*	(2.1-4.3)	1.7*	(1.1-2.8)
Finished primary	1.5*	(1.2-2.0)	1.2	(1.0-1.4)	2.0*	(1.4-2.8)	1.1	(0.7-1.8)
Some secondary	1.4*	(1.1-1.7)	1.2*	(1.1-1.3)	1.6*	(1.3-2.0)	1.0	(0.8-1.4)
Finished secondary	1.0	(0.8-1.2)	1.1	(1.0-1.2)	1.3*	(1.1-1.6)	0.8	(0.6-1.0)
Some college	1.0	(0.8-1.2)	1.1	(1.0-1.2)	1.3*	(1.0-1.6)	0.8	(0.6-1.0)
Finished college	1.0		1.0		1.0		1.0	
Education level difference^d^	χ^2^_6_ = 33.6*, *P* < .001		χ^2^_6_ = 16.2*, *P* = 0.013		χ^2^_6_ = 54.1*, *P* < .001		χ^2^_6_ = 14.8*, *P* = 0.022	
Household income								
Low	1.4*	(1.2-1.7)	1.1*	(1.0-1.2)	1.6*	(1.3-1.9)	1.4*	(1.0-1.8)
Low-average	1.3*	(1.0-1.5)	1.0	(0.9-1.1)	1.4*	(1.1-1.7)	1.3	(1.0-1.7)
High-average	1.1	(0.9-1.3)	1.0	(0.9-1.1)	1.1	(0.9-1.4)	1.1	(0.9-1.4)
High	1.0		1.0		1.0		1.0	
Household income difference^d^	χ^2^_3_ = 19.4*, *P* < .001		χ^2^_3_ = 10.5*, *P* = 0.015		χ^2^_3_ = 23.1*, *P* < .001		χ^2^_3_ = 6.9, *P* = 0.077	
N^e^	142,405		6,081,561		5758		3460	

*Significant at the .05 level, 2 sided test

^a^These estimates are based on logistic regression models adjusted for age, gender and country

^b^These estimates are based on survival models adjusted for age-cohorts, gender, person-years and country

^c^These estimates are based on logistic regression models adjusted for time since social anxiety disorder onset, age of social anxiety disorder onset, gender and country

^d^Chi square test of significant differences between blocks of sociodemographic variables

^e^Denominator N: 142,405 = total sample; 6,081,561 = number of person-years in the survival models; 5,758 = number of lifetime cases of social anxiety disorder; 3,460 = number of 12-month social anxiety disorder cases

### Comorbidity

Table 6 shows that respondents with either lifetime or 12-month SAD are most likely to meet lifetime criteria for other anxiety disorders (59.8% and 64.9%), less likely to meet lifetime criteria for mood and substance use disorders, and least likely to meet lifetime criteria for impulse control disorders (19.3% and 21.9%); in both cases around 80% of such respondents meet lifetime criteria for any other mental disorder. Similarly, respondents with 12-month SAD are most likely to meet 12-month criteria for other anxiety disorders (52.7%), less likely to meet 12-month criteria for mood and impulse control disorders, and least likely to meet 12-month criteria for substance use disorders (10.2%); with 66.9% of such respondents meeting 12-month criteria for any other disorder. For both lifetime and 12-month SAD, SAD begins earlier in only 31.4–35.4% of cases of anxiety disorder, but SAD begins earlier in 48.8–80.9% of cases of mood disorder, substance use disorder, or impulse control disorder.

**Table 6:** Comorbidity of SAD with other DSM-IV disorders

	SAD cases with comorbid disorders									
	Mood disorder^a^		Anxiety disorder^b^		Impulse control disorder^c^		Substance use disorder^d^		Any mental disorder^e^	
	%	SE	%	SE	%	SE	%	SE	%	SE
Lifetime comorbidity^f^										
Lifetime	47.0	1.0	59.8	1.0	19.3	0.8	26.7	0.8	78.8	0.8
12-month	49.8	1.2	64.9	1.2	21.9	1.1	27.0	1.0	81.8	1.0
12-month comorbidity^g^										
12-month	33.4	1.1	52.7	1.2	12.7	0.9	10.2	0.7	66.9	1.2
Temporal priority of SAD^h^										
Lifetime	71.8	1.1	35.4	1.2	49.8	2.3	80.9	1.3	40.4	1.1
12-month	69.1	1.5	31.4	1.4	48.8	2.3	79.7	1.6	35.2	1.2

^a^Respondents with major depressive episode or bipolar disorder (broad)

^b^Respondents with panic disorder, generalized anxiety disorder, specific phobia, agoraphobia, post-traumatic stress disorder, or separation anxiety disorder

^c^Respondents with intermittent explosive disorder, conduct disorder, attention deficit disorder, oppositional defiant disorder, binge eating disorder, or bulimia nervosa

^d^Respondents with alcohol abuse with or without dependence or drug abuse with or without dependence

^e^Respondents with any disorder listed above

^f^Percentage of respondents with either lifetime or 12-month SAD who also meet lifetime criteria for at least one of the other DSM-IV disorders

^g^The human services sector or complementary and alternative medicine (CAM) sector

^h^Percentage of respondents with either lifetime or 12-month SAD and at least one of the other disorders, whose age of onset of SAD is reported to be younger than the age of onset of all comorbid disorders under consideration (i.e., either mood, anxiety, substance use, impulse control, or any disorder)

*SE* standard error

### Treatment

Among those with 12-month SAD, the percentage reporting treatment of any kind (i.e., specialty mental health, general medical care, health care, human services, complementary and alternative medicine, non-health care) in the past 12 months differs significantly by impairment, with 38% receiving any treatment (Table 7). Across all countries, any treatment is lowest in those with moderate impairment (27.4%), and highest in those with severe impairment (46.9%). This pattern holds true for specialty mental health, general medical care, and health care, but human services, complementary and alternative medicine, and non-health care are most commonly used by those with mild impairment. Treatment rates for those with any impairment are lowest in low/lower-income countries (18.0%), and highest in high income countries (44.2%). This pattern holds true for cases with any impairment across all treatment sectors, and for almost all treatment sectors across different levels of impairment.

**Table 7:** Among those with 12-month SAD, percent reporting treatment in the past 12 months by Sheehan impairment severity

Sector of treatment	Sheehan Disability Scale (SDS) category^a^											
	Mild impairment			Moderate impairment			Severe impairment			Any impairment		
	(Score: 1–3)			(Score: 4–6)			(Score: 7–10)					
	%	SE	Comparison between countries^b^	%	SE	Comparison between countries^b^	%	SE	Comparison between countries^b^	%	SE	Comparison between countries^b^
Specialty mental health^c^												
Low/lower-middle income	10.7	6.0	χ^2^ = 1.4, *P* = 0.25	5.2	2.4	χ^2^ = 3.4*, *P* = 0.03	6.3	2.3	χ^2^ = 33.4*, *P* < 0.001	7.8	1.9	χ^2^ = 32.6*, *P* < 0.001
Upper-middle income	13.9	4.2		12.4	2.6		15.3	3.0		13.2	1.7	
High income	19.2	2.0		12.6	1.4		34.4	1.7		23.3	0.9	
All countries combined	17.5	1.7		11.7	1.1		27.7	1.4		19.8	0.8	
General medical^d^												
Low/lower-middle income	–	–	χ^2^ = 14.4*, *P* < 0.001	9.9	3.7	χ^2^ = 5.1*, *P* = 0.01	7.0	2.4	χ^2^ = 44.8*, *P* < 0.001	7.8	1.7	χ^2^ = 65.6*, *P* < 0.001
Upper-middle income	13.8	3.9		12.3	3.1		15.0	2.8		13.7	1.7	
High income	28.8	2.2		20.9	1.9		39.0	1.9		30.8	1.1	
All countries combined	23.9	1.8		17.8	1.5		31.0	1.5		25.2	0.9	
Health care^e^												
Low/lower-middle income	12.4	6.0	χ^2^ = 8.6*, *P* < 0.001	15.0	4.1	χ^2^ = 3.3*, *P* = 0.04	12.7	3.2	χ^2^ = 43.7*, *P* < 0.001	14.5	2.6	χ^2^ = 54.3*, *P* < 0.001
Upper-middle income	23.6	4.6		22.6	3.4		26.0	3.6		23.7	1.9	
High income	36.7	2.3		26.7	2.0		54.6	1.9		40.9	1.1	
All countries combined	32.0	2.0		24.5	1.6		44.6	1.6		34.9	0.9	
Human services^f^												
Low/lower-middle income	–	–	χ^2^ = 5.1*, *P* = 0.01	–	–	χ^2^ = 0.3, *P* = 0.76	3.5	1.7	χ^2^ = 5.1*, *P* = 0.01	3.4	1.3	χ^2^ = 2.5, *P* = 0.08
Upper-middle income	4.5	2.4		4.8	2.0		2.3	1.2		3.6	1.1	
High income	7.7	1.6		3.5	0.8		7.1	1.0		5.7	0.5	
All countries combined	6.5	1.2		3.9	0.8		5.8	0.7		5.1	0.5	
CAM^g^												
Low/lower-middle income	–	–	–	–	–	χ^2^ = 14.3*, *P* < 0.001	–	–	χ^2^ = 12.5*, *P* < 0.001	1.6	0.6	χ^2^ = 26.9*, *P* < 0.001
Upper-middle income	–	–		2.7	1.4		2.5	1.2		2.3	0.7	
High income	9.1	1.7		5.2	0.9		8.5	1.0		7.8	0.6	
All countries combined	7.3	1.3		4.0	0.7		6.6	0.8		6.1	0.5	
Non-health care^h^												
Low/lower-middle income	–	–	χ^2^ = 6.3*, *P* < 0.001	–	–	χ^2^ = 0.2, *P* = 0.80	4.7	1.8	χ^2^ = 11.9*, *P* < 0.001	4.5	1.4	χ^2^ = 15.1*, *P* < 0.001
Upper-middle income	5.0	2.4		7.3	2.3		4.7	1.7		5.6	1.3	
High income	13.7	1.9		7.6	1.1		13.6	1.3		11.7	0.7	
All countries combined	11.3	1.5		7.2	1.0		11.0	1.0		9.8	0.6	
Any treatment^i^												
Low/lower-middle income	15.9	6.1	χ^2^ = 9.2*, *P* < 0.001	20.2	4.9	χ^2^ = 2.0, *P* = 0.13	15.3	3.4	χ^2^ = 44.5*, *P* < 0.001	18.0	2.7	χ^2^ = 52.3*, *P* < 0.001
Upper-middle income	26.6	4.7		24.6	3.5		27.2	3.7		25.7	2.1	
High income	41.8	2.5		29.5	2.0		57.1	1.9		44.2	1.1	
All countries combined	36.6	2.1		27.4	1.7		46.9	1.6		38.0	1.0	

*Significant at the 0.05 level

A dash was inserted for low cell counts (<5 cases)

^a^Highest severity category across four SDS role domains

^b^Chi-square test of homogeneity to determine if there is variation in prevalence of treatment estimates across countries. Chi-square test is only generated where there is more than one stable cell (> = 5 cases) for each combination of treatment sector and Sheehan impairment

^c^The mental health specialist sector, which includes psychiatrist and non-psychiatrist mental health specialists (psychiatrist, psychologist, or other non-psychiatrist mental health professional; social worker or counselor in a mental health specialty setting; use of a mental health helpline; or overnight admissions for a mental health or drug or alcohol problems, with a presumption of daily contact with a psychiatrist)

^d^The general medical sector (general practitioner, other medical doctor, nurse, occupational therapist, or any health care professional)

^e^The mental health specialist sector or the general medical sector

^f^The human services sector (religious or spiritual advisor or social worker or counselor in any setting other than a specialty mental health setting)

^g^The CAM (complementary and alternative medicine) sector (any other type of healer such as herbalist or homeopath, participation in an Internet support group, or participation in a self-help group)

^h^The human services sector or CAM

^i^Respondents who sought any form of professional treatments listed in the footnotes above

## Discussion

A number of limitations of the current study deserve mention. A first important issue is that of sampling. Response rates differ widely across the WMH surveys [12]; while response rates do not appear to be related to SAD prevalence, it is possible that in some settings, particularly those where treatment is less available, those with the most severe SAD were unable to participate in surveys. Surveys also differed in their focus; some included only metropolitan areas, while others employed nationally representative samples; such differences may also have affected prevalence estimates. The surveys also excluded a range of respondents, including institutionalized patients, and people who were too intoxicated to be interviewed. Finally, samples in the WMH surveys also reflected survivor bias; given the 10- to 15-year gap in life expectancy between those in lower and higher income countries, this may also affect prevalence estimates [23]. Taken together, the prevalence rates provided here are therefore conservative. It is also relevant to note that only two African countries were studied, limiting conclusions about distinctions across geographic regions.

Second, the measure of SAD used in the WMH surveys has important limitations. The CIDI relies on a screening section that employs relatively few stem questions, and this may lead to under-estimation of SAD in some settings (as noted, there is no stem question that addresses the symptom of offending others, which is thought to characterize social anxiety in some cultures, and which is now captured in the DSM-5 diagnostic criteria for SAD) [24–27]. Furthermore, no attempt was made to develop distinct cut-off points for SAD in different countries or to go beyond the DSM-IV criteria to develop distinct criteria for different countries that might have increased detection of SAD. It is relevant to emphasize that in countries where blinded clinical reappraisal interviews were undertaken, there was no evidence for systematic bias in the diagnostic threshold for SAD [18]. However, clinical reappraisal interviews were carried out in only a subset of WMH countries, and it is possible that such studies would have found systematic differences in CIDI sensitivity and specificity across contexts.

Bearing in mind these limitations, the WMH surveys provide unique cross-national data on SAD, and are able to address a number of questions about this disorder. Some cross-national differences in SAD epidemiology are apparent: SAD 30-day, 12-month, and lifetime prevalence are lowest in low/lower-middle income countries and in the African and Eastern Mediterranean regions, highest in upper-middle income countries and the Americas and the Western Pacific regions, and there are some differences in domains of role impairment and in treatment rates across country, income region, and WHO region. Crucially, however, there are a number of consistent patterns across the globe: SAD has an early age of onset, is a persistent disorder, and is associated with specific socio-demographic features (younger age, female sex, unmarried status, lower education, and lower income) and with similar patterns of comorbidity and health care utilization.

A previous cross-national study indicated that SAD prevalence differs across different countries, with lifetime prevalence estimates ranging from 0.5 in Korea to 2.6 in the USA [8]. However, that survey was done in only four countries, and assessed only three social fears as part of the simple phobia section of the Diagnostic Interview Schedule. The current data extend such work with surveys across a broad range of countries, and with a comprehensive assessment of SAD. Differences in prevalence across countries continue to be observed, as is the case for other common mental disorders in the WMH surveys. Such differences may reflect artifactual variation across surveys (for example, mental disorder stigma may be higher in lower income settings, resulting in decreased willingness to self-disclose, and an under-estimation of prevalence) or cross-national differences in underlying mechanisms relevant to pathogenesis (for example, greater access to greater social capital and more community engagement in lower income countries).

However, the finding here of similar proportions of SAD respondents with any severe role impairment across country income and geographic groupings suggests that differences in prevalence are not simply due to regional differences in diagnostic thresholding. In higher income countries and in particular regions of the globe such as the Americas, Western Pacific, and Western Europe, there is a higher prevalence of SAD, and SAD is associated with more impairment in the social domain than in other domains, suggesting high demands for social performance in such contexts. The persistence of SAD as well as proportion with any role impairment are highest in upper-middle income countries, Africa, and the Eastern Mediterranean, perhaps pointing to growing performance demands in these regions, but with fewer treatment resources than in higher income countries. The disjunction between lower prevalence but higher persistence of SAD in particular regions may be valuable in suggesting hypotheses, such as this one, about relevant causal mechanisms in SAD.

Our findings that SAD epidemiology demonstrates similar patterns across the globe, being associated with early age of onset, impairment in multiple domains, characteristic socio-demographic correlates (younger age, female gender, unmarried status, lower education, lower household income), and particular patterns of mental disorder comorbidity, again confirms and extends previous work. Thus, for example, we were able to demonstrate that across the globe SAD disorder persistence is particularly highly associated with lower education, episode persistence is particularly associated with being a student, while both disorder and episode persistence are associated with being a homemaker. While it has previously been demonstrated that SAD more likely follows other anxiety disorders, and precedes depression [1], here we provide novel data on the comorbidity of SAD with impulse control disorders; this is valuable given that a link between social anxiety and aggression has been posited in the animal and clinical literature [28, 29]. It is notable that in both lifetime and 12-month SAD, SAD begins earlier in only 31.4–35.4% of cases of comorbid anxiety disorder, due to the common comorbidity with specific phobia which has the earliest onset of the anxiety disorders, but SAD begins earlier in 48.8–80.9% of cases of comorbid mood disorder, substance use disorder, or impulse control disorder. We also provide novel data on treatment rates; these are highest where impairment is most severe and in countries with higher income.

## Conclusions

In conclusion, data from the WMH survey provide the most comprehensive picture of the global epidemiology of SAD to date and help address the key question of whether this condition is a peculiarly Western construct. There are apparent differences in SAD prevalence and domains of role impairment across the globe, with further work needed to delineate more rigorously the reasons for such differences and to investigate possible mechanisms relevant to understanding them. Nevertheless, the data indicate that across the world, SAD is a prevalent condition that is characterized by early age of onset, as well as disorder and episode persistence. Furthermore in low, middle, and high income countries, as well as in a range of geographic regions, SAD is associated with specific socio-demographic correlates (younger age, female gender, unmarried status, lower education, lower household income), particular comorbidity patterns (typically beginning later than specific phobia, but earlier than other anxiety disorders, mood, substance use, or impulse control disorders), and common patterns of health care utilization. Taken together, these cross-national data emphasize the international clinical and public health significance of SAD.

## Appendix 1

**Table 8:** Days out of role due to 12-month SAD by role impairment

Sheehan disability domain	Days out of role due to 12-month social anxiety phobia^b^						
	Severe (Score: 7-10)			Not severe (Score: 0-6)			*F* test, *P* value^c^
	Number (*n*)	Mean	SE	Number (*n*)	Mean	SE	
Home	2010	12.8	1.2	355	92.6	8.8	75.9*, *P* < 0.001
Work	1804	8.4	0.9	536	84.0	6.9	116.6*, *P* < 0.001
Relationship	1629	11.4	1.5	743	54.2	4.7	67.5*, *P* < 0.001
Social	1472	9.4	1.3	901	49.8	4.1	82.6*, *P* < 0.001
Any^a^	1183	4.5	0.7	1193	45.4	3.5	124.3*, *P* < 0.001

*Significant at the 0.05 level

^a^Mean days out of role presented for subgroups of respondents defined by their highest severity category across the four Sheehan disability domains (home, work, relationship, and social)

^b^Mean (SE) days out of role due to 12-month SAD: 24.7 (1.8) days

^c^Bivariate linear regression to test for significant differences in severity. No controls were used

## Appendix 2

**Table 9:** Bivariate associations between socio-demographics correlates and DSM-IV SAD (low/lower-middle income countries)

Correlates	30-day SAD^a^		Lifetime SAD^b^		12-month SAD among lifetime cases^c^		30-day SAD among 12-month cases^c^	
	OR	(95% CI)	OR	(95% CI)	OR	(95% CI)	OR	(95% CI)
Age-cohort								
18–29	6.7*	(2.9–15.5)	7.1*	(4.3–11.6)				
30–44	4.8*	(2.1–11.0)	4.7*	(2.8–7.7)				
45–59	3.6*	(1.4–8.9)	3.2*	(1.9–5.5)				
60+	1.0		1.0					
Age-cohort difference^d^	χ^2^_3_ = 26.6*, *P* < 0.001		χ^2^_3_ = 74.7*, *P* < 0.001					
Age of onset								
Early					2.7*	(1.3–5.5)	1.3	(0.6–2.8)
Early-average					1.4	(0.8–2.4)	1.4	(0.7–3.2)
Late-average					1.1	(0.6–1.9)	1.4	(0.7–2.8)
Late					1.0		1.0	
Age of onset difference^d^					χ^2^_3_ = 9.3*, *P* = 0.026		χ^2^_3_ = 1.3, *P* = 0.721	
Time since onset (continuous)					0.98*	(0.96–0.99)	0.99	(0.97–1.02)
					χ^2^_1_ = 7.4*, *P* = 0.007		χ^2^_1_ = 0.3, *P* = 0.573	
Gender								
Female	1.4	(0.9–2.1)	1.1	(0.9–1.4)	1.5	(1.0–2.2)	1.1	(0.7–1.9)
Male	1.0		1.0		1.0		1.0	
Gender difference^d^	χ^2^_1_ = 2.8, *P* = 0.092		χ^2^_1_ = 1.3, *P* = 0.255		χ^2^_1_ = 3.7, *P* = 0.055		χ^2^_1_ = 0.2, *P* = 0.679	
Employment status								
Student	1.4	(0.6–3.1)	1.1	(0.6–1.8)	1.7	(0.6–4.8)	3.7*	(1.3–10.8)
Homemaker	1.4	(0.8–2.4)	1.0	(0.8–1.4)	1.7	(0.9–3.2)	1.3	(0.6–3.0)
Retired	0.7	(0.3–1.9)	1.9	(1.0–3.8)	0.6	(0.2–1.5)	0.7	(0.3–2.2)
Other	1.0	(0.6–1.7)	1.0	(0.7–1.3)	2.4*	(1.2–4.9)	0.8	(0.4–1.7)
Employed	1.0		1.0		1.0		1.0	
Employment status difference^d^	χ^2^_4_ = 2.7, *P* = 0.606		χ^2^_4_ = 4.2, *P* = 0.387		χ^2^_4_ = 9.9*, *P* = 0.043		χ^2^_4_ = 7.4, *P* = 0.115	
Marital status								
Never married	1.1	(0.7–1.7)	1.4*	(1.1–1.8)	0.8	(0.5–1.3)	1.0	(0.5–1.8)
Divorced/separated/widowed	1.3	(0.8–2.1)	1.3	(0.9–1.8)	1.0	(0.5–2.1)	1.2	(0.5–2.7)
Currently married	1.0		1.0		1.0		1.0	
Marital status difference^d^	χ^2^_2_ = 0.8, *P* = 0.664		χ^2^_2_ = 7.4*, *P* = 0.025		χ^2^_2_ = 1.0, *P* = 0.613		χ^2^_2_ = 0.2, *P* = 0.927	
Education level								
No education	1.2	(0.4–3.1)	0.9	(0.5–1.8)	–	–	1.1	(0.3–3.6)
Some primary	1.4	(0.6–3.2)	0.9	(0.5–1.4)	1.5	(0.6–4.2)	2.8	(0.9–8.9)
Primary finished	1.9	(0.8–4.1)	1.1	(0.7–1.8)	1.6	(0.7–3.7)	2.0	(0.7–6.2)
Some secondary	1.2	(0.5–2.5)	0.9	(0.6–1.3)	1.5	(0.7–3.3)	1.0	(0.5–2.4)
Secondary finished	0.9	(0.5–1.7)	1.0	(0.7–1.4)	1.5	(0.8–2.9)	0.8	(0.4–1.6)
Some college	0.6	(0.3–1.0)	0.8	(0.6–1.2)	1.1	(0.5–2.1)	0.5	(0.2–1.3)
College finished	1.0		1.0		1.0		1.0	
Education level difference^d^	χ^2^_3_ = 12.2, *P* = 0.058		χ^2^_3_ = 2.6, *P* = 0.856		χ^2^_3_ = 3.5, *P* = 0.739		χ^2^_3_ = 8.9, *P* = 0.181	
Household income								
Low	0.9	(0.5–1.6)	0.9	(0.6–1.2)	1.2	(0.6–2.3)	1.2	(0.6–2.6)
Low-average	1.2	(0.6–2.1)	0.9	(0.7–1.3)	2.0*	(1.0–4.1)	0.9	(0.4–2.1)
High-average	0.9	(0.5–1.5)	0.9	(0.7–1.2)	1.8	(0.9–3.6)	0.7	(0.4–1.4)
High	1.0		1.0		1.0		1.0	
Household income difference^d^	χ^2^_3_ = 1.4, *P* = 0.709		χ^2^_3_ = 1.0, *P* = 0.800		χ^2^_3_ = 5.9, *P* = 0.117		χ^2^_3_ = 2.4, *P* = 0.498	
*N* ^e^	36,498		1,426,232		564		349	

*Significant at the 0.05 level, two-sided test

^a^These estimates are based on logistic regression models adjusted for age, gender, and low/lower-middle income countries

^b^These estimates are based on survival models adjusted for age-cohorts, gender, person-years, and low/lower-middle income countries

^c^These estimates are based on logistic regression models adjusted for time since SAD onset, age of SAD onset, gender, and low/lower-middle income countries

^d^Chi-square test exploring significant differences between blocks of socio-demographic variables

^e^Denominator *N*: 36,498 = total sample; 1,426,232 = number of person-years in the survival model; 564 = number of lifetime cases of SAD; 349 = number of 12-month cases of SAD

A dash was inserted for low cell counts (<5 cases)

## Appendix 3

**Table 10:** Bivariate associations between socio-demographics correlates and DSM-IV SAD (upper-middle income countries)

Correlates	30-day SAD^a^		Lifetime risk of SAD^b^		12-month SAD among lifetime cases^c^		30-day SAD among 12-month cases^c^	
	OR	(95% CI)	OR	(95% CI)	OR	(95% CI)	OR	(95% CI)
Age-cohort								
18–29	2.0*	(1.2–3.1)	3.9*	(2.7–5.5)				
30–44	2.1*	(1.3–3.3)	2.9*	(2.1–4.0)				
45–59	1.9*	(1.2–3.1)	2.1*	(1.5–3.1)				
60+	1.0		1.0					
Age-cohort difference^d^	χ^2^_3_ = 9.8*, *P* = 0.021		χ^2^_3_ = 70.3*, *P* < 0.001					
Age of onset								
Early					1.0	(0.6–2.0)	0.5*	(0.3–1.0)
Early-average					0.8	(0.5–1.4)	0.6	(0.3–1.1)
Late-average					0.9	(0.5–1.5)	1.0	(0.6–1.7)
Late					1.0		1.0	
Age of onset difference^d^					χ^2^_3_ = 1.0, *P* = 0.810		χ^2^_3_ = 6.4, *P* = 0.092	
Time since onset (continuous)					0.99	(0.98–1.01)	1.02*	(1.00–1.03)
					χ^2^_1_ = 1.0, *P* = 0.309		χ^2^_1_ = 5.8*, *P* = 0.016	
Gender								
Female	2.0*	(1.6–2.6)	1.5*	(1.3–1.8)	1.6*	(1.1–2.4)	1.6*	(1.0–2.3)
Male	1.0		1.0		1.0		1.0	
Gender difference^d^	χ^2^_1_ = 29.5*, *P* < 0.001		χ^2^_1_ = 19.6*, *P* < 0.001		χ^2^_1_ = 4.7*, *P* = 0.030		χ^2^_1_ = 4.5*, *P* = 0.034	
Employment status								
Student	1.8	(1.0–3.3)	1.4	(0.9–2.1)	0.6	(0.3–1.5)	–	–
Homemaker	1.3	(1.0–1.8)	1.1	(0.8–1.3)	1.4	(0.8–2.3)	1.8*	(1.1–3.1)
Retired	0.4*	(0.2–0.9)	0.7	(0.5–1.0)	1.0	(0.4–2.7)	0.4	(0.1–1.3)
Other	1.4	(1.0–2.0)	1.3	(1.0–1.6)	1.0	(0.6–1.8)	1.2	(0.7–2.1)
Employed	1.0		1.0		1.0		1.0	
Employment status difference^d^	χ^2^_4_ = 15.8*, *P* = 0.003		χ^2^_4_ = 7.8, *P* = 0.101		χ^2^_4_ = 3.9, *P* = 0.414		χ^2^_4_ = 18.7*, *P* = 0.001	
Marital status								
Never married	1.0	(0.7–1.3)	1.3*	(1.0–1.7)	1.0	(0.6–1.6)	0.8	(0.5–1.2)
Divorced/separated/widowed	1.1	(0.8–1.6)	1.3*	(1.0–1.7)	1.0	(0.6–1.7)	0.8	(0.4–1.4)
Currently married	1.0		1.0		1.0		1.0	
Marital status difference^d^	χ^2^_2_ = 0.4, *P* = 0.815		χ^2^_2_ = 8.4*, *P* = 0.015		χ^2^_2_ = 0.0, *P* = 0.996		χ^2^_2_ = 1.5, *P* = 0.484	
Education level								
No education	1.4	(0.7–2.9)	0.7	(0.4–1.2)	–	–	2.1	(0.6–7.4)
Some primary	1.7*	(1.1–2.8)	0.9	(0.7–1.3)	3.8*	(2.0–7.3)	2.2	(0.9–5.3)
Primary finished	1.6	(1.0–2.6)	1.1	(0.8–1.6)	2.4*	(1.1–5.1)	1.3	(0.5–3.3)
Some secondary	1.4	(0.9–2.2)	1.2	(0.9–1.6)	1.3	(0.7–2.6)	1.2	(0.6–2.4)
Secondary finished	1.0	(0.6–1.6)	0.9	(0.7–1.2)	1.1	(0.6–2.0)	1.4	(0.7–2.7)
Some college	1.0	(0.6–1.7)	1.1	(0.8–1.5)	1.5	(0.6–3.8)	0.7	(0.3–1.6)
College finished	1.0		1.0		1.0		1.0	
Education level difference^d^	χ^2^_3_ = 12.9*, *P* = 0.044		χ^2^_3_ = 10.6, *P* = 0.102		χ^2^_3_ = 37.1*, *P* < 0.001		χ^2^_3_ = 9.3, *P* = 0.157	
Household income								
Low	1.2	(0.8–1.7)	1.0	(0.8–1.2)	0.9	(0.5–1.6)	1.6	(0.8–3.1)
Low-average	1.2	(0.8–1.8)	1.0	(0.8–1.3)	1.2	(0.7–2.0)	1.6	(0.9–2.8)
High-average	1.2	(0.9–1.6)	0.8	(0.7–1.1)	1.3	(0.7–2.3)	2.0*	(1.2–3.5)
High	1.0		1.0		1.0		1.0	
Household income difference^d^	χ^2^_3_ = 1.7, *P* = 0.649		χ^2^_3_ = 3.1, *P* = 0.384		χ^2^_3_ = 2.0, *P* = 0.580		χ^2^_3_ = 6.7, *P* = 0.082	
*N* ^e^	28,927		1,206,689		834		601	

*Significant at the 0.05 level, two-sided test

^a^These estimates are based on logistic regression models adjusted for age, gender, and upper-middle income countries

^b^These estimates are based on survival models adjusted for age-cohorts, gender, person-years, and upper-middle income countries

^c^These estimates are based on logistic regression models adjusted for time since SAD onset, age of SAD onset, gender, and upper-middle income countries

^d^Chi-square test exploring significant differences between blocks of socio-demographic variables

^e^Denominator *N*: 28,927 = total sample; 1,206,689 = number of person-years in the survival model; 834 = number of lifetime cases of SAD; 601 = number of 12-month cases of SAD

A dash was inserted for low cell counts (<5 cases)

## Appendix 4

**Table 11:** Bivariate associations between socio-demographics correlates and DSM-IV SAD (high income countries)

Correlates	30-day SAD^a^		Lifetime SAD^b^		12-month SAD among lifetime cases^c^		30-day SAD among 12-month cases^c^	
	OR	(95% CI)	OR	(95% CI)	OR	(95% CI)	OR	(95% CI)
Age-cohort								
18–29	3.3*	(2.7–4.2)	3.3*	(3.0–3.8)				
30–44	2.8*	(2.3–3.5)	2.8*	(2.5–3.2)				
45–59	2.5*	(2.0–3.2)	2.3*	(2.1–2.7)				
60+	1.0		1.0					
Age-cohort difference^d^	χ^2^_3_ = 119.5*, *P* < 0.001		χ^2^_3_ = 414.6*, *P* < 0.001					
Age of onset								
Early					1.5*	(1.1–1.9)	1.0	(0.8–1.4)
Early-average					1.5*	(1.2–1.9)	0.9	(0.7–1.3)
Late-average					1.2	(0.9–1.4)	0.9	(0.7–1.1)
Late					1.0		1.0	
Age of onset difference^d^					χ^2^_3_ = 13.4*, *P* = 0.004		χ^2^_3_ = 1.8, *P* = 0.615	
Time since onset (Continuous)					0.98*	(0.97–0.98)	1.01	(1.00–1.01)
					χ^2^_1_ = 57.0*, *P* < 0.001		χ^2^_1_ = 2.9, *P* = 0.087	
Gender								
Female	1.6*	(1.4–1.8)	1.3*	(1.2–1.4)	1.3*	(1.1–1.5)	1.2	(1.0–1.4)
Male	1.0		1.0		1.0		1.0	
Gender difference^d^	χ^2^_1_ = 38.8*, *P* < 0.001		χ^2^_1_ = 44.7*, *P* < 0.001		χ^2^_1_ = 8.5*, *P* = 0.004		χ^2^_1_ = 2.8, *P* = 0.097	
Employment status								
Student	1.3	(1.0–1.8)	1.1	(0.9–1.4)	1.2	(0.8–1.8)	1.5	(1.0–2.3)
Homemaker	1.4*	(1.2–1.8)	1.2*	(1.1–1.4)	1.3*	(1.0–1.7)	1.3	(0.9–1.8)
Retired	0.7*	(0.5–1.0)	0.8*	(0.7–1.0)	1.1	(0.8–1.5)	1.1	(0.7–1.6)
Other	2.2*	(1.8–2.7)	1.6*	(1.5–1.9)	2.3*	(1.7–3.1)	1.0	(0.7–1.4)
Employed	1.0		1.0		1.0		1.0	
Employment status difference^d^	χ^2^_4_ = 81.7*, *P* < 0.001		χ^2^_4_ = 84.1*, *P* < 0.001		χ^2^_4_ = 34.4*, *P* < 0.001		χ^2^_4_ = 5.3, *P* = 0.258	
Marital status								
Never married	1.3*	(1.1–1.6)	1.4*	(1.3–1.6)	1.5*	(1.2–1.8)	1.0	(0.8–1.3)
Divorced/separated/widowed	1.6*	(1.3–2.0)	1.3*	(1.2–1.5)	1.5*	(1.2–1.8)	1.1	(0.9–1.4)
Currently married	1.0		1.0		1.0		1.0	
Marital status difference^d^	χ^2^_2_ = 30.0*, *P* < 0.001		χ^2^_2_ = 63.3*, *P* < 0.001		χ^2^_2_ = 26.0*, *P* < 0.001		χ^2^_2_ = 0.7, *P* = 0.709	
Education level								
No education	–	–	–	–	–	–	–	–
Some primary	1.9*	(1.1–3.1)	1.2	(1.0–1.6)	2.9*	(1.7–5.0)	1.5	(0.7–3.1)
Primary finished	1.3	(0.8–1.9)	1.2	(1.0–1.5)	1.7*	(1.0–2.9)	0.9	(0.5–1.5)
Some secondary	1.4*	(1.1–1.8)	1.2*	(1.1–1.4)	1.7*	(1.3–2.1)	1.0	(0.7–1.4)
Secondary finished	1.0	(0.8–1.2)	1.1	(1.0–1.2)	1.3*	(1.1–1.6)	0.8	(0.6–1.0)
Some college	1.1	(0.9–1.4)	1.2*	(1.0–1.3)	1.3*	(1.0–1.7)	0.8	(0.6–1.1)
College finished	1.0		1.0		1.0		1.0	
Education level difference^d^	χ^2^_3_ = 17.2*, *P* = 0.009		χ^2^_3_ = 15.6*, *P* = 0.016		χ^2^_3_ = 29.4*, *P* < 0.001		χ^2^_3_ = 7.6, *P* = 0.268	
Household income								
Low	1.6*	(1.3–2.0)	1.2*	(1.1–1.4)	1.8*	(1.4–2.2)	1.3	(1.0–1.8)
Low-average	1.3*	(1.0–1.6)	1.0	(0.9–1.2)	1.4*	(1.1–1.7)	1.3	(1.0–1.8)
High-average	1.1	(0.9–1.4)	1.1	(1.0–1.2)	1.1	(0.9–1.4)	1.0	(0.8–1.4)
High	1.0		1.0		1.0		1.0	
Household income difference^d^	χ^2^_3_ = 25.1*, *P* < 0.001		χ^2^_3_ = 15.9*, *P* = 0.001		χ^2^_3_ = 32.7*, *P* < 0.001		χ^2^_3_ = 5.7, *P* = 0.126	
*N* ^e^	76,980		3,448,640		4360		2510	

*Significant at the 0.05 level, two-sided test

^a^These estimates are based on logistic regression models adjusted for age, gender, and high income countries

^b^These estimates are based on survival models adjusted for age-cohorts, gender, person-years, and high income countries

^c^These estimates are based on logistic regression models adjusted for time since SAD onset, age of SAD onset, gender, and high income countries

^d^Chi-square test exploring significant differences between blocks of socio-demographic variables

^e^Denominator *N*: 76,980 = total sample; 3,448,640 = number of person-years in the survival model; 4360 = number of lifetime cases of SAD; 2510 = number of 12-month cases of SAD

A dash was inserted for low cell counts (<5 cases)
